# The Immunomodulatory Activity of Jacaric Acid, a Conjugated Linolenic Acid Isomer, on Murine Peritoneal Macrophages

**DOI:** 10.1371/journal.pone.0143684

**Published:** 2015-12-02

**Authors:** Wai Nam Liu, Kwok Nam Leung

**Affiliations:** Biochemistry Programme, School of Life Sciences, The Chinese University of Hong Kong, Shatin, HKSAR, China; INSERM, FRANCE

## Abstract

This study aims at demonstrating the immunomodulatory property of jacaric acid, a conjugated linolenic acid (CLNA) isomer that is present in jacaranda seed oil, on murine peritoneal macrophages. Our results showed that jacaric acid exhibited no significant cytotoxicity on the thioglycollate-elicited murine peritoneal macrophages as revealed by the neutral red uptake assay, but markedly increased their cytostatic activity on the T-cell lymphoma MBL-2 cells as measured by the fluorometric CyQuant^®^ NF Cell Proliferation Assay Kit. Flow cytometric analysis indicated that jacaric acid could enhance the endocytic activity of macrophages and elevated their intracellular production of superoxide anion. Moreover, jacaric acid-treated macrophages showed an increase in the production of nitric oxide which was accompanied by an increase in the expression level of inducible nitric oxide synthase protein. In addition, the secretion of several pro-inflammatory cytokines, including interferon-γ, interleukin-1β and tumor necrosis factor-α, was up-regulated. Collectively, our results indicated that the naturally-occurring CLNA isomer, jacaric acid, could exhibit immunomodulating activity on the murine peritoneal macrophages *in vitro*, suggesting that this CLNA isomer may act as an immunopotentiator which can be exploited for the treatment of some immunological disorders with minimal toxicity and fewer side effects.

## Introduction

Conjugated fatty acids (CFA) are a mixture of the positional and geometric isomers of polyunsaturated fatty acids (PUFA) with conjugated double bonds, in which two carbon-carbon double bonds are separated solely by one carbon-carbon single bond [1]. The most common and naturally-occurring CFA include conjugated linoleic acids (CLA) and conjugated linolenic acids (CLNA). Previous reports indicated that CLA can be found in meat and dairy products of ruminant animals [2] while CLNA occur in different plant seed oils [3]. CLA have been studied most extensively in view of their diverse metabolic and physiological effects [4,5] while CLNA have received increasing attention in recent years due to their relative abundance (up to 30–70% of total lipids) in some plant seed oils [3,6]. Previous *in vitro* and *in vivo* studies have shown that CLNA exhibit pleiotropic physiological and pharmacological activities, including anti-inflammatory, anti-obese, anti-carcinogenic and immunomodulatory properties [7,8]. An earlier report showed that consumption of punicic acid (9Z, 11E, 13Z-CLNA isomer) that is present in pomegranate seed oil could enhance the function of B-cells, which play a central role in humoral immune response [9]. It was found that the splenocytes isolated from C57BL/6N mice fed with 0.12% or 1.2% pomegranate seed oil produced larger amounts of IgG and IgM, the major immunoglobulins involved in the antigenic response mediated by the B-cells. In addition, Ike et al. (2005) reported that α-eleostearic acid (9Z, 11E, 13E-CLNA isomer) found in bitter gourd could induce IFN-γ production in mice treated with heat-inactivated *Propionibacterium acnes*, suggesting that T-helper 1 (Th1) cellular immunity, which is essential for preventing intracellular parasitic infection, could be activated by α-eleostearic acid [10]. A review of the current literature has shown that different CLA isomers (9Z, 11E-CLA isomer and 10E, 12Z-CLA isomers) are capable of modulating the functional properties of monocytes and macrophages [11]. For instance, McClelland et al. (2010) demonstrated that CLA isomers can inhibit the migration of human monocytic THP-1 cells and primary blood monocytes, and reduce the inflammatory output of the LPS-activated THP-1 macrophages [12]. In a recent study, Gaetano et al. (2015) showed that the 9Z, 11E-CLA isomer can inhibit the production of pro-inflammatory cytokines in human macrophages, and primed human monocytes towards an anti-inflammatory MΦ2 phenotype [13]. These studies started to shed some lights on the immunomodulatory activities of CLA on macrophages, nevertheless, the modulatory effects of CLNA on murine primary macrophages remain poorly understood.

In the present study, we found that jacaric acid (8Z, 10E, 12Z-CLNA isomer) could activate murine peritoneal macrophages by enhancing their cytostatic and endocytic activities, production of reactive oxygen and nitrogen species, and the secretion of various pro-inflammatory cytokines, while exhibiting minimal cytotoxicity to these cells.

## Materials and Methods

### Chemicals and reagents

**Fig 1:**
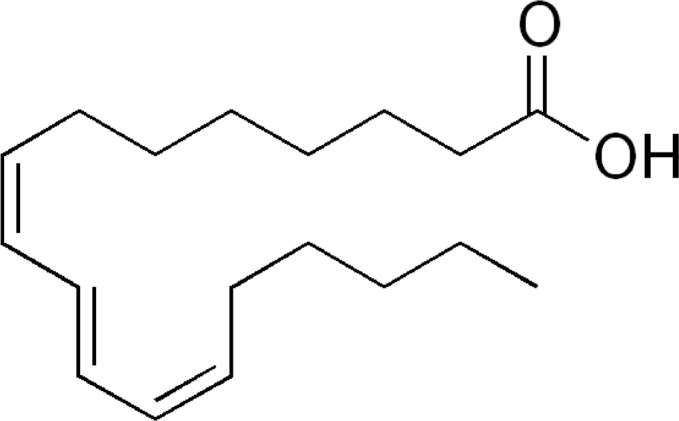
Chemical structure of jacaric acid (8Z, 10E, 12Z-CLNA isomer).

Jacaric acid (8Z, 10E, 12Z-CLNA isomer; Fig 1) used in the study, with an estimated purity >97%, was purchased from Larodan Fine Chemicals AB, Sweden. The stock solution (0.2 M) was prepared by dissolving the powder in cell culture-tested ethanol (Sigma-Aldrich Co., USA). The endotoxin concentrations in the stock solution and the working solution of jacaric acid were estimated by ToxinSensor^TM^ Chromogenic LAL Endotoxin Assay Kit (GenScript Inc., USA) to be 0.175 ± 0.007 ng/ml and 0.104 ± 0.001 ng/ml, respectively, using the procedures according to the manufacturer’s instructions. All other chemicals, unless otherwise stated, were purchased from Sigma-Aldrich.

### Culture of cell line

MBL-2 cell line is a Moloney virus-induced T cell lymphoma of C57BL/6J mice [14], which was purchased from the American Type Culture Collection (ATCC) in USA. The cells were maintained in RPMI-1640 medium (Gibco, USA) supplemented with 10% fetal bovine serum (FBS; Gibco) and 1% antibiotics (100 U/ml penicillin G, 100 μg/ml streptomycin sulfate and 0.25 μg/ml amphotericin B in 0.85% saline from Gibco) in a humidified incubator containing 5% CO_2_ at 37°C.

### Mice

Inbred female BALB/c (H-2^d^) mice aged 6 to 8 weeks old were obtained from the Laboratory Animal Services Centre of the Chinese University of Hong Kong and were kept in a specific pathogen-free condition. The animal experiments were conducted with the licence under Animals (Control of Experiments) Ordinance (Cap.340) issued by the Department of Health of the Hong Kong Government, and according to the guidelines of the Animal Experimentation Ethics Committee, The Chinese University of Hong Kong. The Animal Experimentation Ethics Committee of the Chinese University of Hong Kong had specifically approved this study on May 13, 2013 with the approval Ref. No. 13/040/GRF.

### Preparation of murine peritoneal macrophages and assay for cell viability

Thioglycollate (TG)-elicited peritoneal macrophages were isolated from BALB/c mice as previously described with slight modifications [15]. Briefly, BALB/c mice were each injected intraperitoneally with 2.5 ml of sterile 3% TG broth solution (Difco Laboratories Inc., USA) to elicit an inflammatory response and to recruit the peritoneal macrophages. Three days later, mice were sacrificed by cervical dislocation and peritoneal exudate cells (PEC) were harvested and resuspended in ammonium chloride potassium (ACK) lysis buffer for erythrocyte lysis. Subsequently, the PEC were centrifuged at 400 xg for 5 min, washed with PBS three times and maintained in RPMI medium supplemented with 10% fetal bovine serum and 1% antibiotics in a humidified incubator containing 5% CO_2_ at 37°C.

In our experiments, the PEC (2.5 x 10^6^ /ml) were seeded in a flat-bottomed 96-well microtiter plate at 37°C for 3 h to allow the adherence of macrophages to the bottom of the wells. After incubation, non-adherent cells were removed and washed with warm RPMI medium three times. The adhering macrophages were incubated with different concentrations of jacaric acid (0 to 100 μM) at 37°C for 72 h. The cell viability was measured by the standard neutral red uptake assay as described previously [16].

### Assay of macrophage-mediated cytostatic activity

This was carried out as described previously by Leung et al. [17]. Briefly, the peritoneal macrophages seeded in a 96-well plate were pre-treated with different concentrations of jacaric acid (either 50 or 100 μM) in the absence or presence of lipopolysaccharides (LPS) (60 ng/ml) at 37°C for 72 h for activation [18]. Cells treated with 0.1% ethanol acted as the control.

For the measurement of macrophage-mediated cytostatic activity towards tumor cells, jacaric acid and LPS were removed by three washes with warm RPMI medium, and 10^4^ MBL-2 cells were added to each well in a final volume of 200 μl. After incubation at 37°C for 48 h, the cytostatic activity of the jacaric acid-treated macrophages was measured by using the CyQuant^®^ NF Cell Proliferative Assay Kit (Molecular Probes; Invitrogen Corp., USA) and recorded using a fluorescence plate reader (Tecan Polarion, USA) as described previously [19]. The percentage growth inhibition of MBL-2 cells induced by jacaric acid-treated macrophages was calculated as follows:

$$\%\;\text{inhibition}=\;\left[1-\;\left(\frac{\text{Fluorescence intensity of jacaric acid}-\text{treated macrophages}}{\text{Fluorescence intensity of ethanol}-\text{treated macrophages}}\right)\right]\times \;100$$

To determine whether the jacaric acid-treated macrophages could also elicit the growth-inhibitory effect without direct contact with the MBL-2 cells, jacaric acid and LPS were first removed by three washes with warm RPMI medium, and complete RPMI medium (without MBL-2 cells) was then added to each well in a final volume of 200 μl. After incubation at 37°C for 48 h, the cell-free supernatant was transferred to another 96-well plate seeded with 10^4^ MBL-2 cells. The anti-proliferative effect of the supernatant was measured by the cell proliferative assay kit after 48 h incubation at 37°C as described above.

### Assay for endocytic activity of macrophages

The procedures used for the assay of the endocytic activity of macrophages were described previously [20]. In brief, the peritoneal macrophages seeded in a flat-bottomed 6-well microtiter plate were incubated with different concentrations of jacaric acid (either 50 or 100 μM), in the presence of LPS (60 ng/ml) at 37°C for 72 h. Afterwards, the medium was removed and the cells were washed with warm RPMI medium three times. The FITC-conjugated albumin (1 mg/ml) in 1 ml fresh RPMI medium was then added to the cells and further incubated at 37°C for 6 h in dark. Subsequently, the cells were gently removed from the plate by using a sterile cell scraper, centrifuged at 400 xg for 5 min, and resuspended in 1 ml PBS containing 1% (w/v) paraformaldehyde. The endocytic activity of the macrophages was analyzed for FITC fluorescence with an excitation wavelength of 488 nm and an emission wavelength of 525 nm using the FACSCanto^TM^ flow cytometer (BD BioSciences, USA).

### Determination of reactive oxygen species (ROS) production by macrophages

The peritoneal macrophages seeded in a 6-well plate were incubated with different concentrations of jacaric acid (either 50 or 100 μM), in the presence of LPS (60 ng/ml) at 37°C for 72 h. After incubation, the cells were gently removed from the plate, and the cell pellet was stained by 10 μM dihydroethidium (DHE) in 0.5 ml PBS and further incubated at 37°C for 30 min in dark with gentle shaking. Afterwards, the macrophages were analyzed for red fluorescence (FL-3) with an excitation wavelength of 488 nm and an emission wavelength of 660 nm using the FACSCanto^TM^ flow cytometer.

### Measurement of nitric oxide (NO) production by macrophages

The peritoneal macrophages seeded in a 96-well plate were incubated with different concentrations of jacaric acid (either 50 or 100 μM) in the presence of LPS (60 ng/ml) at 37°C for 72 h. After incubation, 100 μl cell-free supernatant from each well was transferred into another 96-well plate, followed by the addition of 100 μl freshly prepared Griess reagent. The plate was incubated at room temperature for 10 min with gentle shaking, and the absorbance at 540 nm was recorded by a Benchmark microplate reader (Bio-Rad Laboratories, USA). The nitrite concentrations of the samples were quantified with reference to the standard sodium nitrite solutions with concentrations ranging from 0 to 64 μM.

### Western blot analysis

Protein expression level was determined by Western blotting technique with the aid of a panel of specific antibodies. Briefly, the peritoneal macrophages seeded in a 6-well plate were incubated with different concentrations of jacaric acid (either 50 or 100 μM) in the presence of LPS (60 ng/ml) at 37°C for 72 h. Cells treated with 0.1% ethanol acted as the control. After incubation, the cell pellet was collected and total protein was extracted by the cell lysis buffer. Protein concentration was measured by Bradford reagent and the protein samples (30 μg/well) were resolved on a 10% polyacrylamide gel and transferred to a PVDF membrane. The membrane was first incubated with the rabbit anti-iNOS antibody (diluted 1:250) (Cell Signaling Technology Inc., USA) or mouse anti-β-actin antibody (diluted 1:2,000) (Santa Cruz Biotechnology), followed by incubation with horseradish peroxidase (HRP)-conjugated secondary antibody (GE Healthcare Limited, UK) and finally developed with the enhanced chemiluminescence reagent (Santa Cruz Biotechnology).

### Assessment of cytokine secretion by ELISA

To determine the secretion of interferon (IFN)-γ, interleukin (IL)-1β and tumor necrosis factor (TNF)-α, the corresponding ELISA kit was used (ExCell Biology, Inc., China). Briefly, the peritoneal macrophages seeded in a 96-well plate were incubated with different concentrations of jacaric acid (either 50 or 100 μM), in the presence of LPS (60 ng/ml) at 37°C for 72 h. The cell-free supernatant was then transferred to another 96-well plate provided in the ELISA kit. Subsequently, the absorbance at 405 nm was measured by the Benchmark microplate reader.

### Statistical analysis

Each experiment was repeated three times and only the results of the most representative experiments were shown. The data were expressed as the arithmetic mean ± standard error (SE). One-way analysis of variance (ANOVA) with *post hoc* Tukey’s Multiple Comparison Test was used for statistical analysis and the differences were considered as statistically significant at *p*<0.05.

## Results

### Jacaric acid induced the cytostatic activity of murine peritoneal macrophages

**Fig 2:**
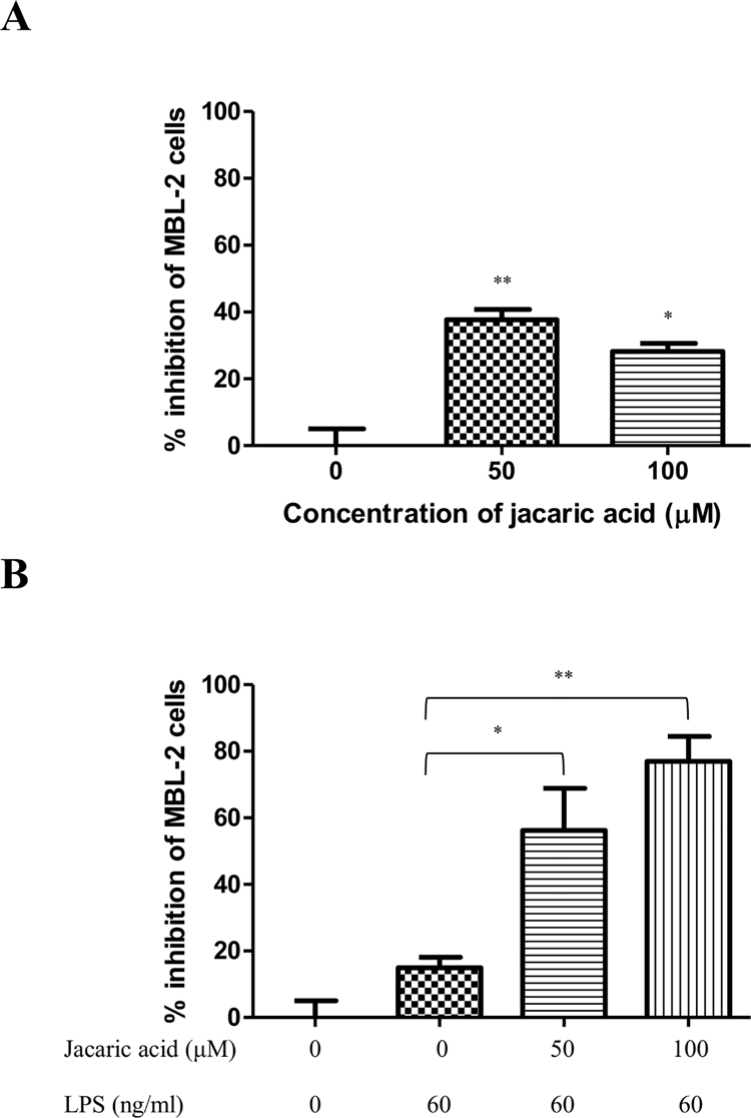
Cytostatic activity of jacaric acid-treated murine peritoneal macrophages on T-cell lymphoma MBL-2 cells. Macrophages were pre-treated with two different concentrations of jacaric acid (either 50 or 100 μM) in the absence (A) or presence (B) of 60 ng/ml LPS at 37°C for 72 h. Cells treated with 0.1% ethanol acted as the control. The pre-treated macrophages were incubated with MBL-2 cells at 37°C for 48 h and the cytostatic activity of macrophages was determined by the CyQuant^®^ NF Cell Proliferation Assay Kit. The results were expressed as the mean percentage inhibition of cell proliferation ± SE. * *p*< 0.05; ** *p*< 0.01.

**Fig 3:**
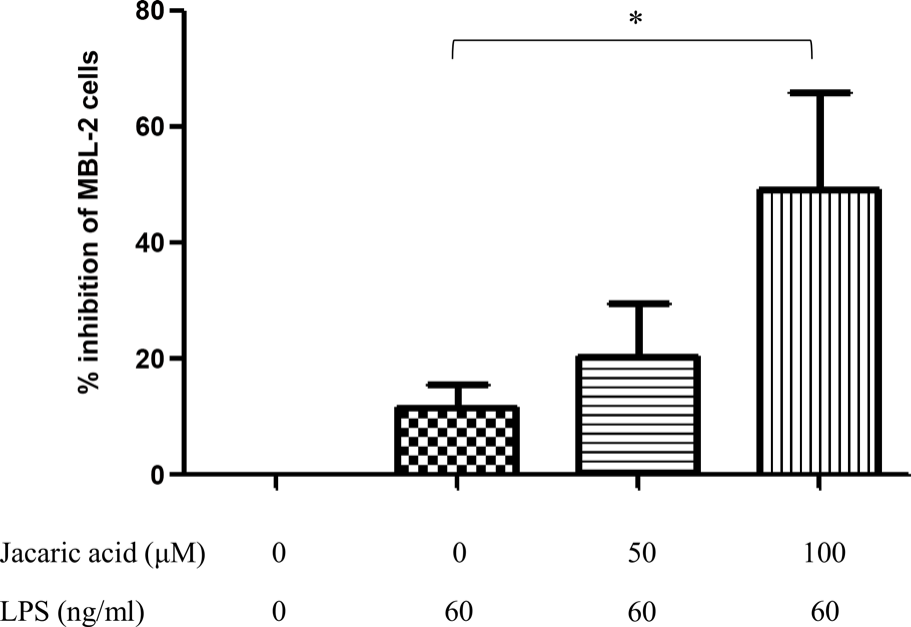
Cytostatic activity of cell-free supernatant from jacaric acid and LPS-treated murine peritoneal macrophages on T-cell lymphoma MBL-2 cells. Macrophages were pre-treated with different concentrations of jacaric acid (either 50 or 100 μM) in the presence of LPS (60 ng/ml) at 37°C for 72 h. Cells treated with 0.1% ethanol acted as the control. The pre-treated macrophages were washed and then incubated with fresh RPMI medium at 37°C for 48 h. Subsequently, the cell-free supernatant was added to the MBL-2 cells and further incubated at 37°C for 48 h. The cytostatic activity of the macrophages was then determined. The results were expressed as the mean percentage inhibition of cell proliferation ± SE. * *p*< 0.05.

To demonstrate the immunomodulatory effects of jacaric acid on macrophages, the cytostatic activity of TG-induced murine peritoneal macrophages against MBL-2 tumor cells was evaluated by the CyQuant^®^ NF Cell Proliferation Assay Kit. As shown in Fig 2A, after pre-treatment with jacaric acid alone in the absence of LPS, the macrophages exhibited cell-to-cell contact-dependent cytostatic effect and inhibited the growth of the MBL-2 cells up to 30–40%. The cytostatic effect was found to be more pronounced in the presence of LPS and it was found that the percentage of inhibition of MBL-2 cell proliferation reached 80% when the macrophages were pre-treated with 100 μM jacaric acid in the presence of LPS (Fig 2B). Apart from the direct cytostatic effect of jacaric acid-treated macrophages on the MBL-2 cells, Fig 3 shows that the cell-free supernatant from the jacaric acid and LPS-treated macrophages also inhibited the growth of the MBL-2 cells in a concentration-dependent manner, but to a lesser extent (around 40%). Remarkably, jacaric acid exhibited little, if any, direct cytotoxicity on the murine peritoneal macrophages, as the percentage of viability of the macrophages remained >90% when the cells were incubated with 100 μM jacaric acid for 72 h (S1 Fig).

### Jacaric acid enhanced the endocytic activity in murine peritoneal macrophages

**Fig 4:**
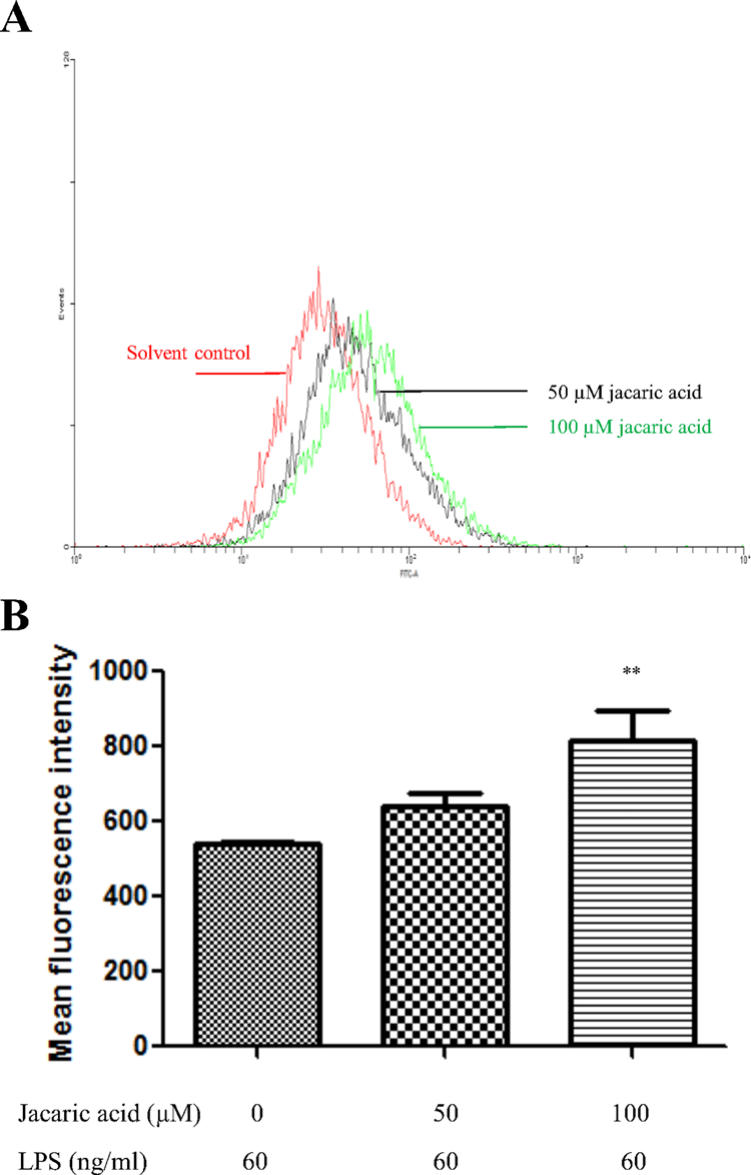
Jacaric acid enhanced the endocytic activity in murine peritoneal macrophages. Macrophages were incubated with different concentrations of jacaric acid (either 50 or 100 μM) in the presence of LPS (60 ng/ml) at 37°C for 72 h. Cells treated with 0.1% ethanol acted as the control. Treated macrophages were further incubated with FITC-conjugated albumin in fresh RPMI medium at 37°C for 6 h in dark. (A) The endocytic activity was analyzed for FITC fluorescence by flow cytometry. (B) The results were expressed as mean ± SE. ** *p*< 0.01.

To determine the possible effects of jacaric acid on other functional properties of macrophages, the endocytic activity of the jacaric acid-treated macrophages was assessed by measuring their ability to endocytose the FITC-conjugated albumin, and the fluorescence uptake was analyzed by flow cytometry. Fig 4A and 4B showed that jacaric acid at 100 μM could enhance the endocytic activity of LPS-treated macrophages, suggesting that the endocytic function of macrophages could be modulated by exposure to jacaric acid *in vitro*.

### Jacaric acid increased ROS and NO generation in murine peritoneal macrophages

**Fig 5:**
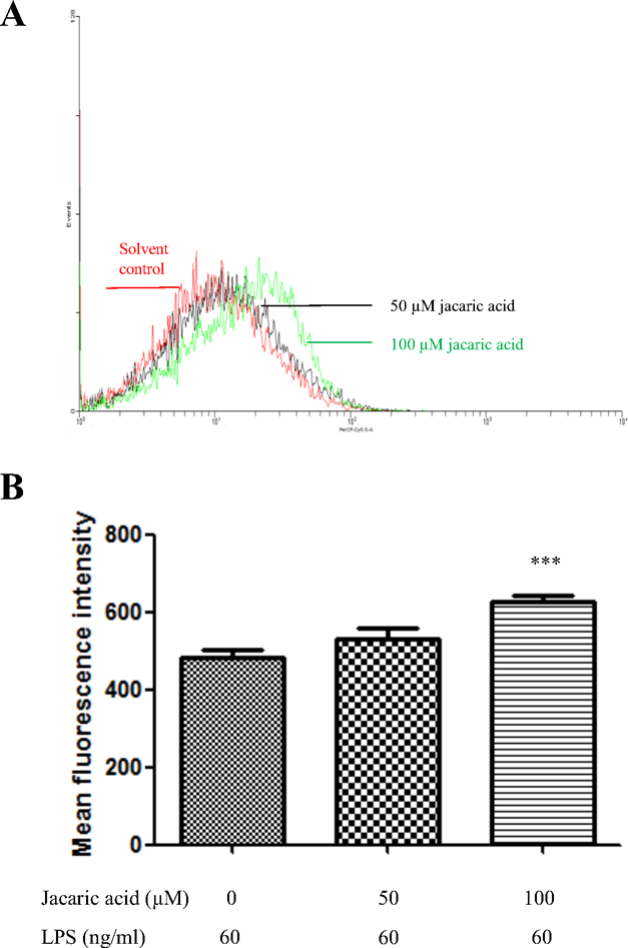
Jacaric acid increased ROS generation in murine peritoneal macrophages. Macrophages were incubated with different concentrations of jacaric acid (either 50 or 100 μM) in the presence of LPS (60 ng/ml) at 37°C for 72 h. Cells treated with 0.1% ethanol acted as the control. (A) The intracellular levels of superoxide anion in the treated cells were measured by staining cells with DHE at 37°C for 30 min and analyzed for red fluorescence (FL-3) by flow cytometry. (B) Results were expressed as mean ± SE. *** *p*< 0.001.

**Fig 6:**
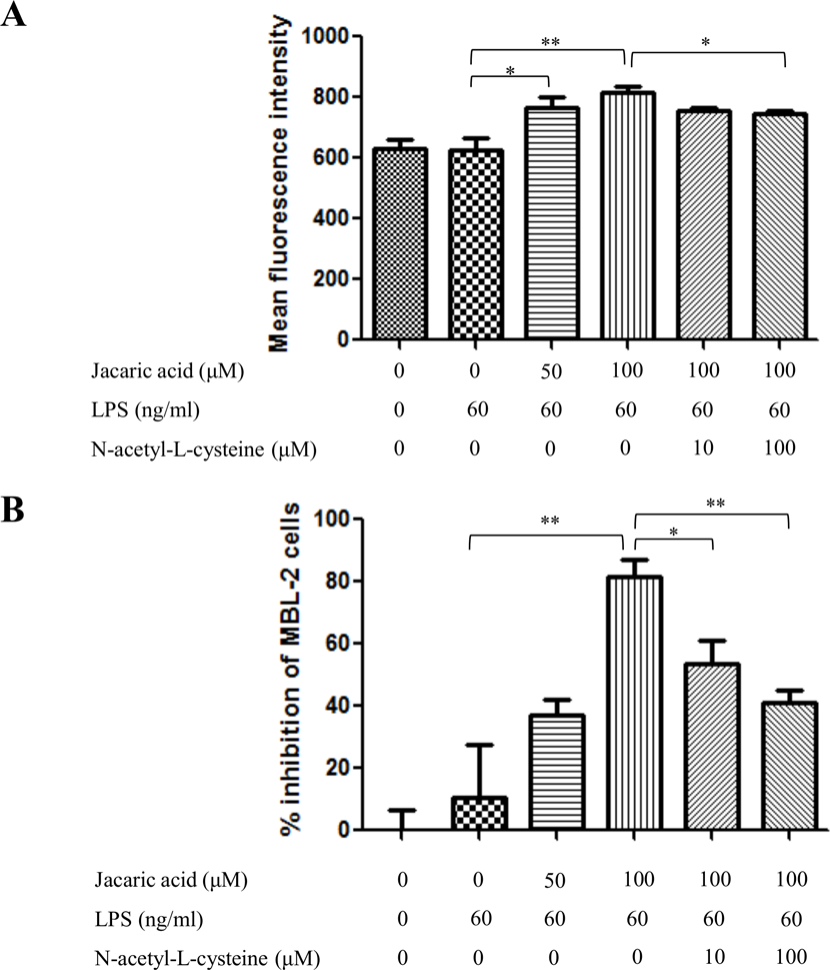
Effect of N-acetyl-L-cysteine on the jacaric acid-induced ROS generation and cytostatic activity in murine peritoneal macrophages. Macrophages were incubated with different concentrations of jacaric acid (either 50 or 100 μM) and N-acetyl-L-cysteine (either 10 or 100 μM), in the presence of LPS (60 ng/ml) at 37°C for 72 h. Cells treated with 0.1% ethanol acted as the control. (A) The intracellular levels of superoxide anion in the treated cells were measured by staining cells with DHE at 37°C for 30 min and analyzed for red fluorescence (FL-3) by flow cytometry. Results were expressed as mean ± SE. * *p*< 0.05; ** *p*< 0.01. (B) The pre-treated macrophages were incubated with MBL-2 cells at 37°C for 48 h and the cytostatic activity of macrophages was determined by the CyQuant^®^ NF Cell Proliferation Assay Kit. The results were expressed as the mean percentage inhibition of cell proliferation ± SE. * *p*< 0.05; ** *p*< 0.01.

It has been shown that the reactive oxygen species and nitric oxide play an important role in cancer killing [21,22]. To determine the changes in the intracellular ROS level, jacaric acid-treated macrophages were stained by DHE for the detection of superoxide anion. Flow cytometric analysis showed that the intracellular level of superoxide anion increased significantly when the macrophages were treated with 100 μM of jacaric acid (Fig 5A and 5B). To test whether that the jacaric acid-induced cytostatic activity in macrophages might be attributed to the increased ROS generation in jacaric acid-treated macrophages, the effect of an exogenous antioxidant, N-acetyl-L-cysteine, on the jacaric acid-induced cytostatic activity in macrophages was examined. As shown in Fig 6A, upon the addition of N-acetyl-L-cysteine, there was a significant decrease in the level of superoxide anion in the jacaric acid-treated macrophages. Moreover, the addition of N-acetyl-L-cysteine could also reduce the cytostatic activity of the jacaric acid-treated macrophages (Fig 6B).

**Fig 7:**
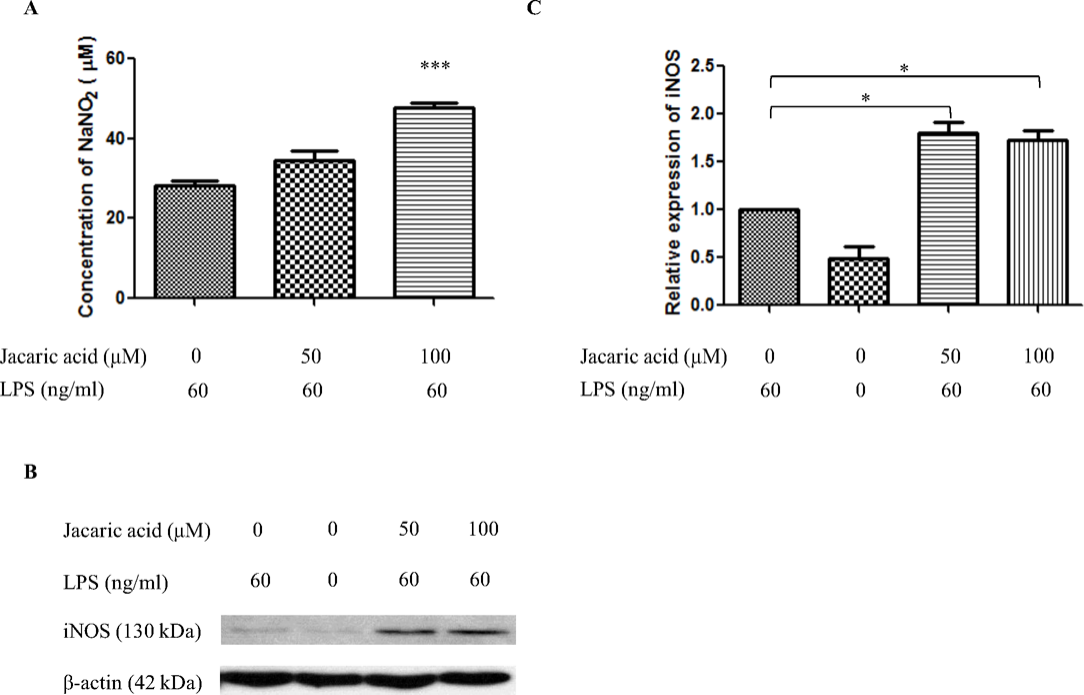
Jacaric acid enhanced NO production in murine peritoneal macrophages. Macrophages were incubated with different concentrations of jacaric acid (either 50 or 100 μM) in the presence of LPS (60 ng/ml) at 37°C for 72 h. Cells treated with 0.1% ethanol acted as the control. (A) Cell-free supernatant was transferred to another 96-well plate and the amount of NO production was detected by Griess reagent. The results were expressed as the mean ± SE. *** *p*< 0.001. (B and C) Protein expression level of iNOS was assayed by Western blotting with β-actin protein as an internal control. The relative protein expression level of iNOS compared to β-actin was quantified. Results represent mean ± SE. * *p*< 0.05.

Apart from the superoxide anion, the production of NO from the murine peritoneal macrophages was measured using the Griess reagent. It was found that jacaric acid could enhance NO production in the murine peritoneal macrophages in a concentration-dependent manner (Fig 7A). To further confirm the modulation of NO production in the macrophages, Western blotting was performed to examine the expression level of iNOS protein (Fig 7B), which is known to be responsible for the production of NO when macrophages are stimulated with activators such as LPS [18]. As shown in Fig 7C, the expression level of iNOS protein increased significantly when the macrophages were treated with 50 μM and 100 μM jacaric acid.

### Jacaric acid enhanced the secretion of pro-inflammatory cytokines in murine peritoneal macrophages

**Fig 8:**
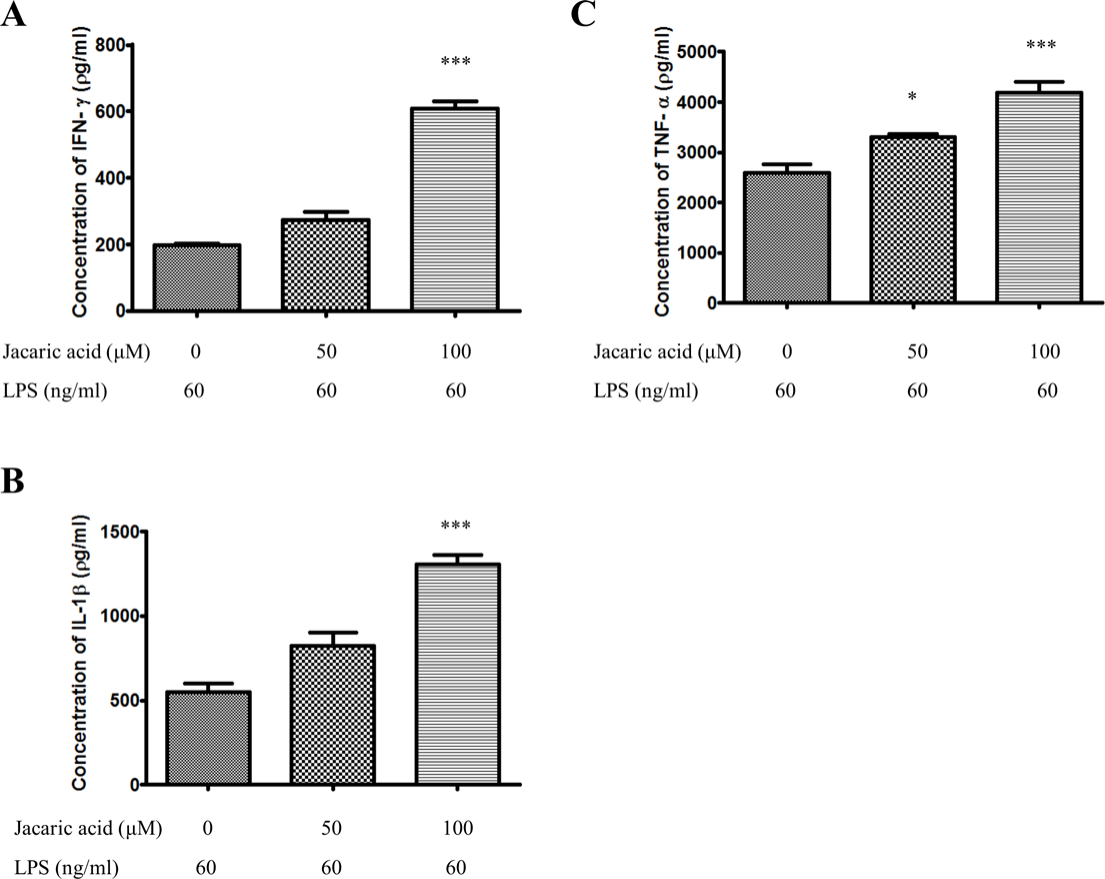
Jacaric acid enhanced the secretion of pro-inflammatory cytokines in murine peritoneal macrophages. Macrophages were incubated with different concentrations of jacaric acid (either 50 or 100 μM) in the presence of LPS (60 ng/ml) at 37°C for 72 h. Cells treated with 0.1% ethanol acted as the control. The concentrations of IFN-γ (A), IL-1β (B) and TNF-α (C) were quantified by ELISA kit according to manufacturers’ instructions, and the results were expressed as mean ± SE. * *p*< 0.05; *** *p*< 0.001.

The effect of jacaric acid on the secretion of several pro-inflammatory cytokines by the murine peritoneal macrophages was assessed by the cytokine-specific ELISA. As shown in Fig 8, the secretion of IFN-γ, IL-1β and TNF-α by the macrophages was increased in a concentration-dependent manner after treatment with jacaric acid. The results clearly indicate that jacaric acid could activate macrophages by enhancing their production of pro-inflammatory cytokines.

## Discussion

Macrophages play a central role in pathophysiological responses, like inflammation, organ healing and regeneration [23]. In addition, macrophages are involved in host defence and the direct destruction of the tumor cells by the release of cytotoxic mediators [24]. These mediators include TNF-α and NO, which can be up-regulated by various immunological stimuli, one of which is LPS [25,26]. Earlier studies by Wallace et al. (2000) suggested that both the amount and type of polyunsaturated fatty acids (PUFA) in the diet of mice affected the production of mediators by macrophages and altered macrophage-mediated cytotoxicity [24]. Ever since then, there are different reports examining the immunomodulatory effects of PUFA such as CLA on the macrophages, and the findings are often inconsistent, depending on both the isomer of CLA and the species of the macrophage cell line used for investigation [11,24]. On one hand, some reports suggested that the 9Z, 11E-CLA and 10E, 12Z-CLA could enhance phagocytosis, upregulate the expression level of TNF-α and increase intracellular ROS synthesis and oxygenation of macrophages [27,28]. In contrast, other studies demonstrated the reduction of NO and TNF-α secretion after treatment with these CLA [29,30]. Moreover, it was found that α-linolenic acid, the non-conjugated counterpart of CLNA, might reduce the generation of ROS in elaidic acid-treated rat peritoneal macrophages [31], Others have shown that 9Z, 11E-CLA might inhibit the production of pro-inflammatory cytokines in human macrophages, and primed human monocytes towards an anti-inflammatory MΦ2 phenotype [13]. In spite of these earlier studies, the modulatory effects of CLNA on macrophages as well as their underlying action mechanisms have not yet been investigated.

In the present study, we found that pre-treatment of murine peritoneal macrophages with jacaric acid, the 8Z, 10E, 12Z-CLNA isomer that is present in jacaranda seed oil, at concentrations higher than 50 μM, in the absence or presence of LPS, could enhance their cytostatic activity on the T-cell lymphoma MBL-2 cells, and the growth-inhibitory effect was found to be more pronounced when there was direct cell-to-cell contact between the LPS- and jacaric acid-treated macrophages, and the tumor target MBL-2 cells. Interestingly, it was found that jacaric acid only exerted slight (<10%) direct cytotoxicity on the macrophages even at higher concentrations up to 100 μM (S1 Fig). Further studies of the macrophage-activating properties of jacaric acid showed that jacaric acid could enhance the endocytic activity of peritoneal macrophages. Similarly, using FITC-conjugated *Escherichia coli*, we found that jacaric acid could also enhance the phagocytic ability of macrophages (S2 Fig). Previous findings from other groups have demonstrated the capability of CLA to enhance phagocytosis through modulation of cyclooxygenase-2 (COX-2) pathway or peroxisome proliferator-activated receptor (PPAR)γ-dependent pathway [27,32]. Nevertheless, whether the COX-2 pathway and PPARγ pathway are involved in the modulatory effect of jacaric acid on the endocytic and phagocytic activities of macrophages require further investigation.

Apart from endocytosis, there might be an increase in the generation of ROS and NO, as these free radicals are reported to play an important role in the lysis of tumor cells [33,34]. Within mammalian cells, a family of nitric oxide synthase (NOS) enzymes has been shown to be involved in NO generation, and three isoforms of the NOS family have been identified, namely endothelial NOS, neuronal NOS and inducible NOS (iNOS) [21,35]. In our present study, it could be seen that there was an increase in the generation of ROS after pre-treatment of the peritoneal macrophages with jacaric acid and LPS. Moreover, the production of NO in the macrophages was elevated, which was accompanied by an increase in the protein expression level of iNOS. Similar results were observed when different CLA isomers were incubated with the human monocytic cell line THP-1 [36], while some reports showed different outcomes [29,30]. The discrepancy in the results might due to a number of reasons, including but not limiting to the origin of the macrophages, the concentrations and the types of CFA used for investigation. Earlier studies have demonstrated the differential effects of dietary fats on the macrophage-mediated cytotoxicity towards tumor cells [24]. Yang et al. (2009) suggested that the oxidative stabilities of various CLA and CLNA isomers were different, depending on the positional and geometric configurations of the isomers, as well as the number of conjugated double bonds in the isomers [37]. This might also account for the discrepancy that different CFA isomers modulated the generation of ROS and NO in macrophages in different ways. In addition, our results showed that jacaric acid-treated peritoneal macrophages significantly stimulated the secretion of pro-inflammatory cytokines, including IFN-γ, IL-1β and TNF-α. It was reported that NO could be generated in macrophages by iNOS following the exposure to these cytokines in the presence of LPS [25]. These results are in line with some *in vitro* and *in vivo* studies [27,38], whereas some reports suggested that CLA did not alter the production of the pro-inflammatory cytokines in human subjects [39,40].

Collectively, our results suggest that jacaric acid is capable of modulating the immune activities of murine macrophages, such as inducing their cytostatic activity on MBL-2 tumor cells, enhancing their endocytic ability, and increasing their production of intracellular ROS and cytokines. The underlying mechanisms for the observed differential immunomodulatory effects of jacaric acid as compared to other CFA such as CLA as shown in earlier studies remain obscure and this is an intriguing aspect that awaits further investigations. Since jacaric acid is found to exert minimal direct cytotoxicity to the murine peritoneal macrophages, especially at lower concentrations, and apparently non-toxic to mice (up to 1 mg/kg/day; S1 Table) [41], further elucidation of the immunomodulatory effects of jacaric acid, both *in vitro* and *in vivo*, may provide better insights for the development of jacaric acid as an immunopotentiator for the treatment of some immunological disorders with minimal toxicity and fewer side effects.

## Supporting Information

S1 Fig Jacaric acid exerted minimal cytotoxicity to the murine peritoneal macrophages. Macrophages seeded in a 96-well plate were incubated with different concentrations of jacaric acid (0–100 μM) at 37°C for 72 h. Cells treated with 0.1% ethanol acted as the control. The viability of the macrophages was determined by neutral red uptake assay. The results were expressed as the mean percentage of cell viability ± SE.
(TIFF)

S2 Fig Jacaric acid enhanced the phagocytic ability of murine peritoneal macrophages. Macrophages seeded in a 96-well plate were incubated with different concentrations of jacaric acid (either 50 or 100 μM) in the presence of LPS (60 ng/ml) at 37°C for 72 h. Cells treated with 0.1% ethanol acted as the control. After incubation, FITC-conjugated *Escherichia coli* (1 mg/ml) were added to the cells and further incubated at 37°C for 1 h in dark. Subsequently, the cells were fixed by 70% ethanol after which propidium iodide (40 μg/ml) was added to the cells. The FITC fluorescence and PI fluorescence were recorded using a fluorescence plate reader, and the results were expressed as the mean phagocytic index (FITC fluorescence/PI fluorescence) ± SE. ** *p*< 0.01.
(TIFF)

S1 Table *In vivo* toxicity test of jacaric acid in BALB/c mice. BALB/c mice in groups of six were fed with different doses of jacaric acid (either 1 mg/kg or 2 mg/kg in 100 μL corn oil) on alternate days from day 0 to day 14. Mice fed with vehicle in 100 μL corn oil acted as the control. The mice were sacrificed on day 14, and the body weight and liver weight were determined by an electronic balance. The results were expressed as average weight ± SE.
(DOCX)
