# Supplementary figures and images for: The Immunomodulatory Activity of Jacaric Acid, a Conjugated Linolenic Acid Isomer, on Murine Peritoneal Macrophages
Source: PLoS One. 2015 Dec 2;10(12):e0143684. doi: 10.1371/journal.pone.0143684 (PMC4667904; doi:10.1371/journal.pone.0143684)

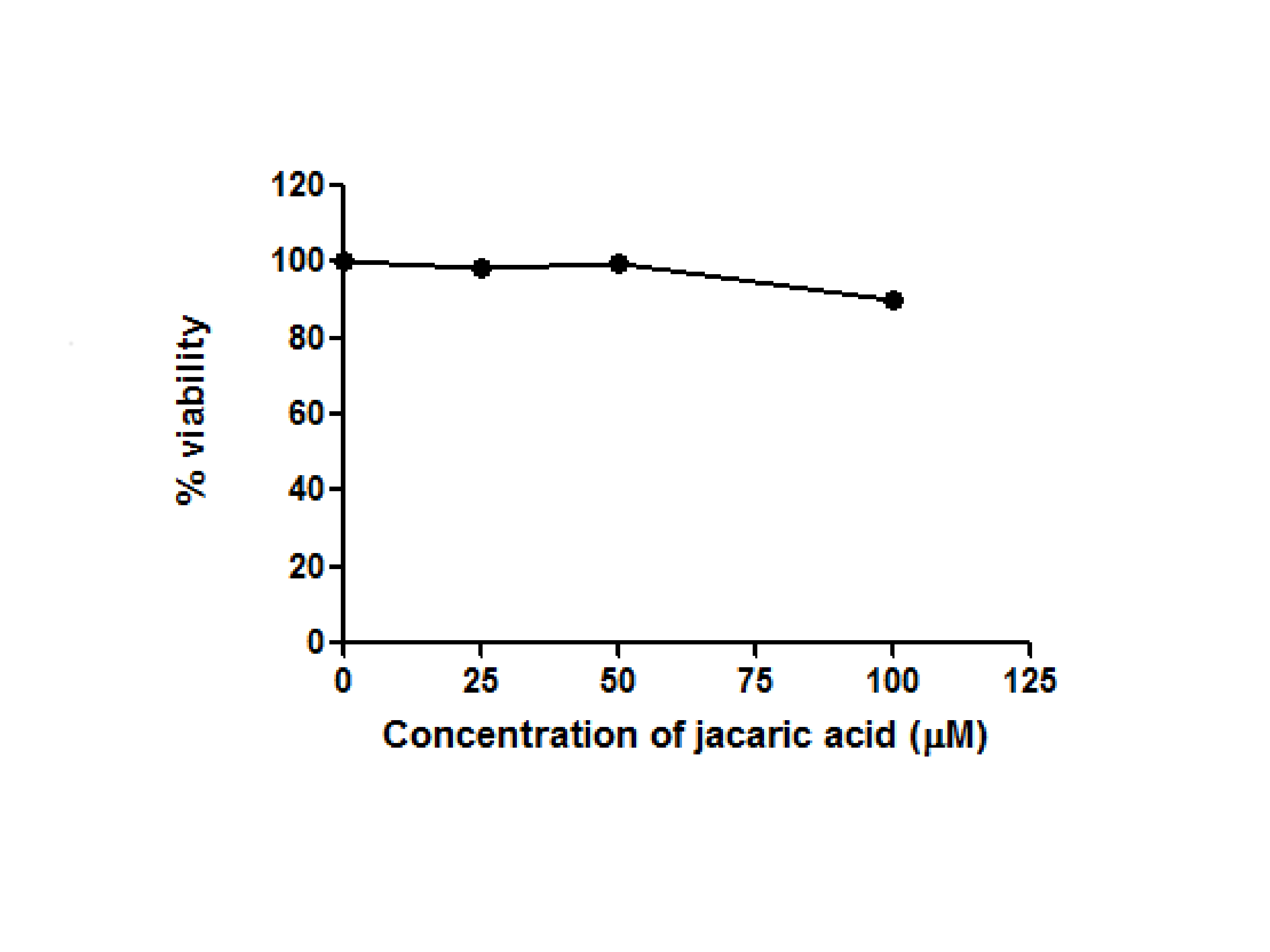

Supplement: S1 Fig — Macrophages seeded in a 96-well plate were incubated with different concentrations of jacaric acid (0–100 μM) at 37°C for 72 h. Cells treated with 0.1% ethanol acted as the control. The viability of the macrophages was determined by neutral red uptake assay. The results were expressed as the mean percentage of cell viability ± SE. (TIFF) [file pone.0143684.s001.tiff]

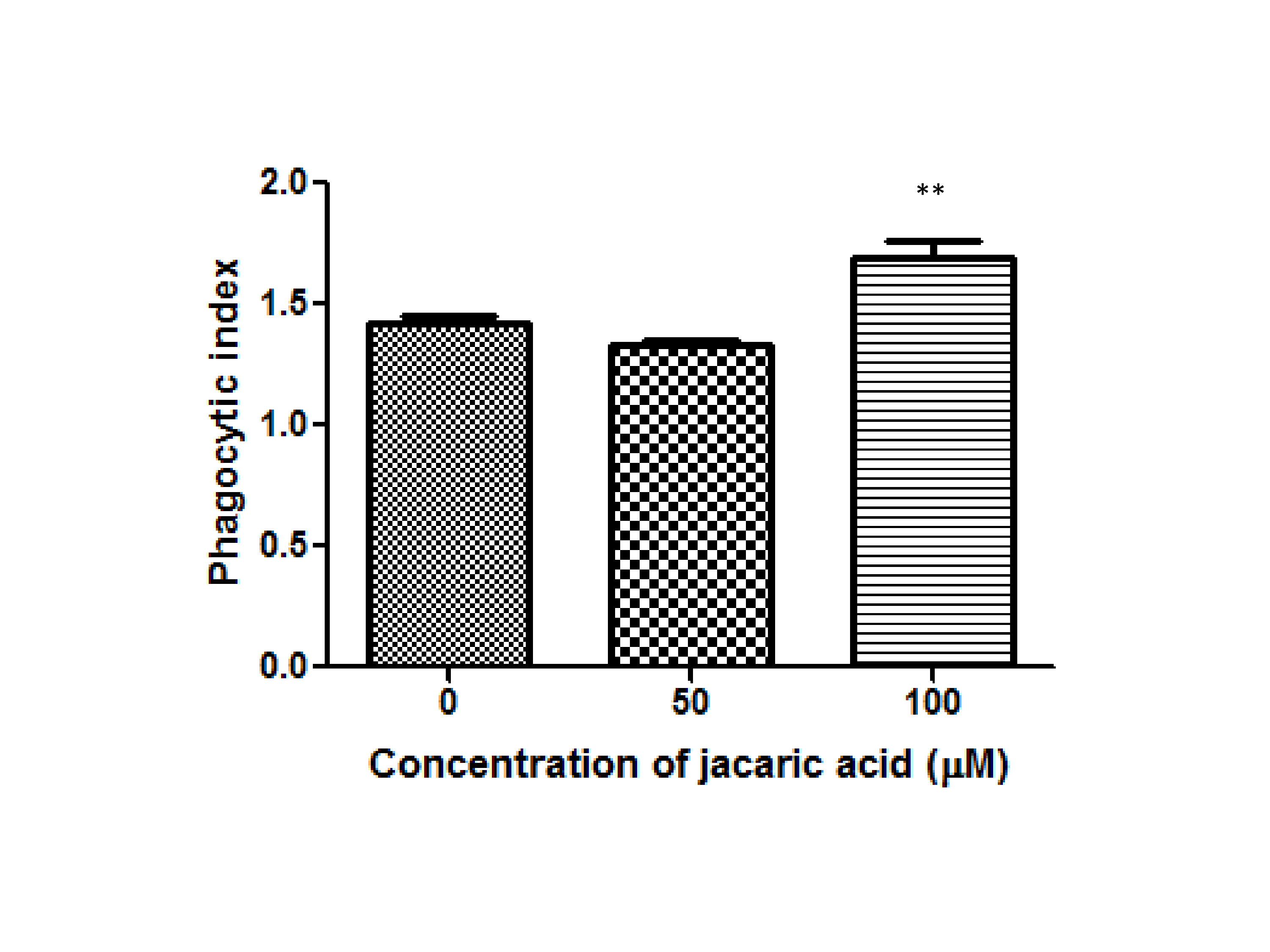

Supplement: S2 Fig — Macrophages seeded in a 96-well plate were incubated with different concentrations of jacaric acid (either 50 or 100 μM) in the presence of LPS (60 ng/ml) at 37°C for 72 h. Cells treated with 0.1% ethanol acted as the control. After incubation, FITC-conjugated Escherichia coli (1 mg/ml) were added to the cells and further incubated at 37°C for 1 h in dark. Subsequently, the cells were fixed by 70% ethanol after which propidium iodide (40 μg/ml) was added to the cells. The FITC fluorescence and PI fluorescence were recorded using a fluorescence plate reader, and the results were expressed as the mean phagocytic index (FITC fluorescence/PI fluorescence) ± SE. ** p< 0.01. (TIFF) [file pone.0143684.s002.tiff]
