# Supplementary material for: The Immunomodulatory Activity of Jacaric Acid, a Conjugated Linolenic Acid Isomer, on Murine Peritoneal Macrophages
Source: PLoS One. 2015 Dec 2;10(12):e0143684. doi: 10.1371/journal.pone.0143684 (PMC4667904; doi:10.1371/journal.pone.0143684)
Supplement: S1 Table — BALB/c mice in groups of six were fed with different doses of jacaric acid (either 1 mg/kg or 2 mg/kg in 100 μL corn oil) on alternate days from day 0 to day 14. Mice fed with vehicle in 100 μL corn oil acted as the control. The mice were sacrificed on day 14, and the body weight and liver weight were determined by an electronic balance. The results were expressed as average weight ± SE. (DOCX) [file pone.0143684.s003.docx]

**Table S1. *In vivo* toxicity test of jacaric acid in BALB/c mice.**

| **Parameters** | **Dose of jacaric acid administered (mg/kg)** | | |
| --- | --- | --- | --- |
|  | **0** | **1** | **2** |
| **Body weight on day 14 (g)** | 18.7 ± 1.63 | 18.7 ± 1.21* | 19.0 ± 1.10* |
| **Liver weight on day 14 (g)** | 1.07 ± 0.23 | 1.00 ± 0.20* | 1.20 ± 0.20* |

*Not significantly different from the control group of mice without jacaric acid administration.
